# Ageing Through the Looking‐Glass: The Different Flavours of Clonal Haematopoiesis

**DOI:** 10.1111/acel.70425

**Published:** 2026-02-22

**Authors:** Jasmine Singh, David J. Curtis, Le Thi Phuong Thao, Erica M. Wood, Zoe K. McQuilten

**Affiliations:** ^1^ School of Public Health and Preventive Medicine Monash University Melbourne Australia; ^2^ Department of Haematology Fiona Stanley Hospital Perth Australia; ^3^ Australian Centre for Blood Diseases Monash University Melbourne Australia; ^4^ Department of Clinical Haematology Alfred Health Melbourne Australia; ^5^ Department of Haematology Monash Health Melbourne Australia

**Keywords:** ageing biomarkers, clonal haematopoiesis, haematopoietic stem cells, inflammageing

## Abstract

Clonal haematopoiesis (CH) is the presence of acquired mutations in blood cells and is a consequence of ageing that is linked to malignancy, cardiovascular disease and other diseases of ageing. CH is a reflection of genomic instability with ageing; however, there is evidence that CH may exacerbate features of normal ageing, including inflammageing and immunosenescence, and more directly contribute to disease causation. CH can manifest as mosaic loss of X or Y, autosomal mosaic chromosomal rearrangements, or point mutations or small insertions or deletions. Until recently, little has been known about the relationship between different forms of CH and other biomarkers of ageing, including whether they are more likely to co‐exist, whether they work synergistically to promote clonal expansion, and whether they have independent impacts on risk of clinical outcomes. Defining the overlap between different forms of CH and other markers of ageing is important to understand the biological processes involved in ageing, and the mechanisms underlying the associations with diseases of ageing. Here we provide an overview of the current literature on intersections of different forms of CH, the clinical implications of these, and a perspective on how CH enhances our understanding of the biology of ageing.

## Graphical abstract

Different forms of CH share common germline and environmental risk factors and have overlapping prevalence and disease associations, suggesting common underlying ageing processes. We explore intersections of different forms of CH, clinical implications of these, and how this enhances our understanding of the biology of ageing.

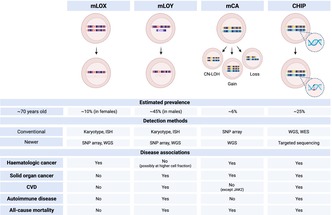

## 1 Introduction

**FIGURE 1:**
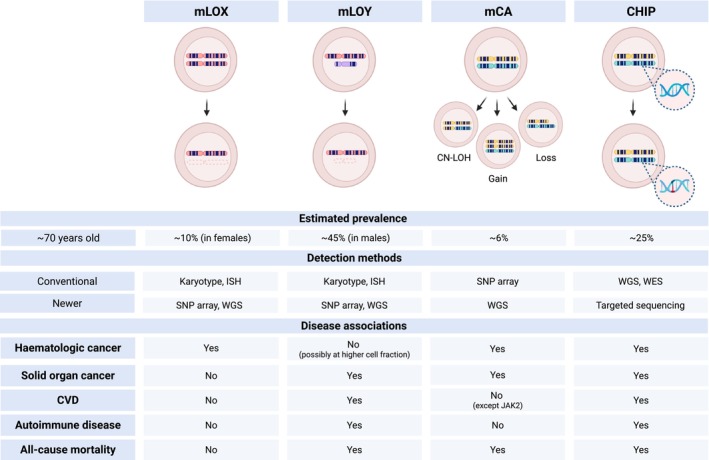
Different forms of clonal haematopoiesis. Clonal haematopoiesis can occur as mLOX, mLOY, mCAs and CHIP. The table compares the prevalence, detection methods (both conventional and more recently described) and disease associations for the different forms of clonal haematopoiesis. Prevalence estimates are influenced by the age of the population and detection methods. In the UK Biobank (which comprises individuals aged 40–69 years with a median age of ~56 years), the reported prevalence is 8% for mLOX (Brown et al. 2023), 20% for mLOY (Thompson et al. 2019), 3%–5% for mCAs (Loh et al. 2018) and 4% for CHIP using whole exome sequencing (Vlasschaert, Mack, et al. 2023). Prevalence increases with age and estimates shown here are for those aged 70 years, and (for CHIP) using targeted sequencing (Cook et al. 2019). Created in https://BioRender.com. CHIP, clonal haematopoiesis of indeterminate potential; CN‐LOH, copy neutral loss of heterozygosity; CVD, cardiovascular disease; ISH, in situ hybridization; mCA, mosaic chromosomal alterations; mLOX, mosaic loss of X; mLOY, mosaic loss of Y; SNP, single nucleotide polymorphism; WES, whole exome sequencing; WGS, whole genome sequencing.

Hallmarks of ageing include chronic inflammation (‘inflammageing’), impaired immune function (immunosenescence), stem cell exhaustion, and genomic instability leading to somatic mosaicism (López‐Otín et al. 2013). Blood is a looking glass for understanding the physiologic changes of ageing, and mechanisms of ageing‐associated diseases. Although clonal haematopoiesis (CH) is a hallmark of blood cancer, it is also now recognised as a feature of ageing in individuals without blood cancers (Genovese et al. 2014; Jaiswal et al. 2014; Loh et al. 2018; Xie et al. 2014). CH can occur in the form of large chromosomal alterations including mosaic loss of X or Y (mLOX or mLOY) and mosaic chromosomal alterations (mCAs) affecting autosomes (Figure 1). More common however, are small insertions or deletions (indels) or point mutations (single nucleotide variants or SNVs), which has been termed clonal haematopoiesis of indeterminate potential (CHIP) when involving myeloid‐cancer associated genes and present at a variant allele frequency (VAF) of at least 2%. In addition to increasing the risk of blood cancers, CHIP and other forms of CH have been associated with various other diseases of ageing (Guo et al. 2020; Lin et al. 2021; Singh et al. 2024; Weeks and Ebert 2023). However, differences in CH definitions, detection methods and cohort characteristics have contributed to heterogeneous and sometimes discordant findings across studies. It has been hypothesised that the different forms of CH may all arise from a ‘common soil’ of genomic instability, that is, that shared heritable and environmental factors may promote the acquisition and subsequent expansion of mutations (Thompson et al. 2019). However, it remains largely unknown whether associations between CH and diseases of ageing reflect correlation or whether CH may directly cause disease. Furthermore, because different methods have been used for detection, there are few cohorts where multiple forms of CH and their implications have been examined. Here, we review the relationship between ageing and CH, including how CH develops, and how it interacts with other features of ageing including inflammageing, immunosenescence, epigenetic ageing and telomere shortening. We also review what is known about the overlap between different forms of CH and whether they make independent contributions to risk of disease.

## 2 Changes in the Haematopoietic System With Ageing

Ageing is associated with a number of changes in the haematopoietic system contributing to increasing prevalence of anaemia, lymphopenia, clonal haematopoiesis and many haematological malignancies, as well as altered immune function. A detailed review of haematopoietic effects of ageing is beyond the scope of this review and has been addressed in several other reviews (Colom Díaz et al. 2023; de Haan and Lazare 2018; Konieczny and Arranz 2018; Shevyrev et al. 2023; Yamashita and Iwama 2022). We provide here an overview of changes in haematopoietic stem cells, mature blood cells and the bone marrow microenvironment with ageing, and the impact of these on immune function including inflammageing and immunosenescence.

**FIGURE 2:**
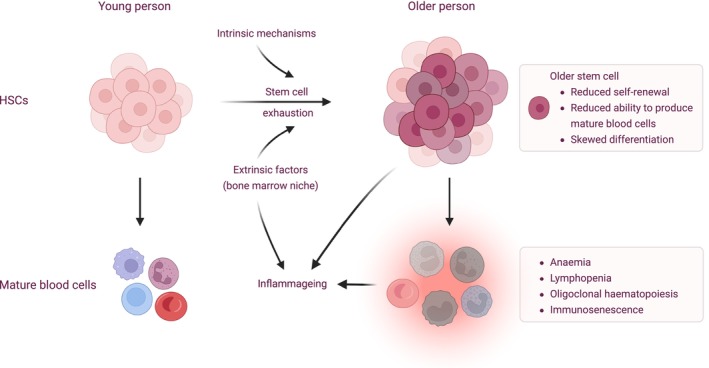
Haematopoietic changes associated with ageing. Ageing is associated with changes in haematopoietic stem cells (HSCs), the bone marrow niche and mature blood cells. Although total numbers of stem cells increase, there is greater heterogeneity in their functional ability. Older stem cells have reduced functional ability, which contributes to anaemia, lymphopenia, immunosenescence and oligoclonal haematopoiesis. Ageing of stem cells is predominantly driven by intrinsic mechanisms; however, extrinsic influences from the bone marrow niche are also important. Haematopoietic consequences of ageing contribute to chronic inflammation (inflammageing) and impaired immune function (immunosenescence). Created in https://BioRender.com.

Haematopoietic stem cells (HSCs) give rise to mature peripheral blood cells via intermediate progenitors. A key hallmark of ageing is stem cell exhaustion, a decline in the functional ability of stem cells (de Haan and Lazare 2018; López‐Otín et al. 2013) (Figure 2). Aged HSCs have reduced self‐renewal ability, as well as reduced ability to produce mature peripheral blood cells and skewed differentiation towards myeloid and platelet lineages. Total numbers of HSCs increase with age (de Haan and Lazare 2018; Dykstra et al. 2011; Morrison et al. 1996), but older individuals have greater functional heterogeneity among HSCs with larger numbers of older HSCs (de Haan and Lazare 2018). Reduced functionality of older HSCs means that mature peripheral blood cells are derived from only a small number of these, resulting in oligoclonal haematopoiesis (Mitchell et al. 2022). Although mutations in HSCs accumulate linearly with age, Mitchell et al. (2022) demonstrated that the switch from polyclonal to oligoclonal haematopoiesis occurs quite abruptly at age 60–70 years, even though the expanded mutations are acquired decades earlier. The precipitous loss of clonal diversity is not explained simply by age‐associated stem cell attrition, but rather appears related to positive selection of clones, which may be due to unidentified intrinsic or extrinsic factors influencing clonal fitness.

Functional changes in HSCs with age are thought to be predominantly mediated by intrinsic mechanisms, as younger HSCs engraft better than older HSCs when transplanted into mice (de Haan and Lazare 2018; Dykstra et al. 2011; Morrison et al. 1996). These mechanisms include accumulation of DNA damage, telomere shortening, increased polarity (asymmetric distribution of specific proteins), impaired autophagy resulting in accumulation of mitochondria and reactive oxygen species, altered epigenetic programming and cellular senescence (de Haan and Lazare 2018; Mejia‐Ramirez and Florian 2020; Shevyrev et al. 2023).

However, HSCs cells also perform better (with better engraftment and less myeloid skewing) when transplanted into younger recipients compared to older recipients (Ergen et al. 2012). Additionally, transplanting aged HSCs into younger mice can at least restore their transcriptional profile although not their regenerative abilities (Kuribayashi et al. 2021). These observations suggest a role for extrinsic influences on HSC ageing as well including exposure to inflammation, infection and age‐associated changes in the bone marrow microenvironment (Colom Díaz et al. 2023; de Haan and Lazare 2018; Ho and Méndez‐Ferrer 2020; Konieczny and Arranz 2018). An important extrinsic influence is the bone marrow niche, which comprises extracellular matrix and various cell types including osteoblasts, mesenchymal stromal cells (MSCs), endothelial cells, adipocytes and others (Colom Díaz et al. 2023; Ho and Méndez‐Ferrer 2020). Changes in the bone marrow niche with ageing include vascular remodelling, altered adrenergic signalling, increased adipogenesis, reduced osteogenic differentiation and accumulation of senescent cells that secrete pro‐inflammatory factors, known as the senescence‐associated secretory phenotype (SASP) (Ho and Méndez‐Ferrer 2020). The pro‐inflammatory microenvironment, whether from cellular senescence or other factors, promotes ageing of HSC. For example, an increase in bone marrow senescent mesenchymal stromal cells (MSCs) and pro‐inflammatory SASP factors has been demonstrated to impair the function of young HSCs (Gnani et al. 2019). Depletion of senescent cells in the bone marrow, for example with the BCL2/Bax inhibitor navitoclax, can restore HSC function in mice (Chang et al. 2016). An increase in the pro‐inflammatory cytokine IL‐1B in the bone marrow niche promotes HSC ageing and blockade of IL‐1B may ameliorate HSC function (Kovtonyuk et al. 2022; Mitchell et al. 2023). The gut microbiome has been implicated as a regulator of haematopoiesis and may contribute to increased IL‐1 with age (Kovtonyuk et al. 2022). Transplant of faecal microbiota from young to aged mice can rejuvenate HSCs (Zeng et al. 2023). Similarly, Ramalingam et al. (2023) recently demonstrated that ageing in mice was associated with reduction in bone marrow niche Netrin‐1 and increased DNA damage. Supplementation of Netrin‐1 was able to reverse DNA damage as well as the regenerative potential of HSCs (Ramalingam et al. 2023). Further studies are needed but it is clear that the pro‐inflammatory microenvironment contributes to HSC ageing, and the concept that targeting external stimuli may be able to prevent or even reverse the effects of ageing is an attractive area of research (Broxmeyer et al. 2020; Geiger et al. 2013; Groarke and Young 2019; Konieczny and Arranz 2018). Recognising the interplay between HSCs and extrinsic influences is also important in understanding how a small number of altered cells (as in clonal haematopoiesis) might be able to exacerbate features of ageing. Recent work at the single cell level suggests that CH mutations in HSCs have both intrinsic and extrinsic effects (Heimlich et al. 2024; Jakobsen et al. 2024). For example, compared to HSCs from individuals without CH, both mutant and non‐mutant HSCs from those with CH are enriched for inflammatory and ageing transcriptomic signatures, but the response to inflammation is attenuated in the mutant compared to non‐mutant cells (Jakobsen et al. 2024).

The ageing haematopoietic system contributes to immunosenescence and inflammageing. Immunosenescence is the age‐related decline in immune‐system function and is characterised by impaired innate and adaptive immunity. Myeloid and platelet skewing associated with ageing HSCs leads to a relative reduction in erythropoiesis and lymphopoiesis, which contribute to lymphopenia as well as anaemia in the elderly. Naïve B and T cells are reduced with less diversity in antigen receptors leading to impaired adaptive immunity (Mogilenko et al. 2022; Nikolich‐Žugich 2018; Shevyrev et al. 2023). Ageing is also associated with impaired function of neutrophils, macrophages and dendritic cells, including impaired phagocytosis and antigen presentation, resulting in impaired innate immunity (Nikolich‐Žugich 2018). Inflammageing is the low grade chronic inflammatory state associated with ageing, and is linked to the pathogenesis of many diseases of ageing (Aunan et al. 2016; Campisi et al. 2019; Konieczny and Arranz 2018). Pro‐inflammatory factors secreted by aged senescent cells, including HSCs, MSCs and mature haematopoietic cells of the immune system, all contribute to inflammageing, and inflammageing in turn leads to further HSC ageing as well as promoting clonal haematopoiesis and malignant transformation (Colom Díaz et al. 2023; Shevyrev et al. 2023).

## 3 Clonal Haematopoiesis

### 3.1 Manifestations of Clonal Haematopoiesis

Clonal haematopoiesis in individuals without haematologic malignancy was first described in the 1960s as loss of a sex chromosome, loss of Y (mLOY) males and loss of X (mLOX) in females (Jacobs et al. 1963). mLOY and mLOX were traditionally detected by karyotyping or in situ hybridisation, but more recent techniques include genotyping arrays, or whole genome sequencing (Barros et al. 2020). During embryonic development in females, one of the two X chromosomes in each cell is randomly inactivated and mLOX preferentially affects the inactive X chromosome (Machiela et al. 2016). Random X chromosome inactivation (XCI) results in mosaicism where approximately 50% of cells express genes from the paternal X chromosome and the other half from the maternal one. Extreme skewing of the maternal: paternal X ratio is a surrogate marker for clonality, and it was demonstrated in the early 1990s that this could occur in healthy women (Busque et al. 1996; Fey et al. 1994; Gale et al. 1991). In 2012, a small number of women with skewed XCI were shown to harbour somatic mutations in the *TET2* gene (Busque et al. 2012). Around the same time, it was recognised that large chromosomal alterations could occur in people without cancer (Jacobs et al. 2012; Laurie et al. 2012). Mosaic chromosomal alterations (mCAs) refer to aneuploidy (either gains or losses), or copy‐neutral loss of heterozygosity (CN‐LOH; also known as uniparental disomy) of large chromosomal regions (Figure 1). In the UK biobank, autosomal mosaicism overall is estimated to occur at a frequency of ~3%–5% (Loh et al. 2018, 2020), compared to X chromosome mosaicism in 8% (Loh et al. 2018) and mosaic loss of Y (mLOY) in up to 20% (Thompson et al. 2019).

In 2014, three large studies highlighted the high prevalence of somatic point mutations and indels in the blood of people without haematological malignancy using whole exome or whole genome sequencing (Genovese et al. 2014; Jaiswal et al. 2014; Xie et al. 2014). Interestingly, such mutations frequently occurred in the same genes that are mutated in myeloid haematological malignancies, and these genes have been referred to as candidate driver or putative driver genes. The World Health Organisation has now defined clonal haematopoiesis of indeterminate potential (CHIP) as the presence of somatic mutations in myeloid cancer‐associated genes, and present at a variant allele frequency (VAF) of ≥ 2%, in the absence of a known haematological malignancy or unexplained cytopenia (Khoury et al. 2022). The presence of clonal abnormalities and cytopenias, without significant morphologic dysplasia, defines the entity of clonal cytopenias of undetermined significance (CCUS) (Arber et al. 2022; Khoury et al. 2022). CCUS has a greater risk of progression to myeloid malignancy than CHIP (Gu et al. 2023; Weeks et al. 2023), and a subset of patients with CCUS have been reported to have outcomes similar to low risk myelodysplastic syndrome (Malcovati et al. 2017; Rossi et al. 2021). Studies of CHIP consistently implicate a restricted set of genes, the most common being the epigenetic modifiers *DNMT3A*, *TET2* and *ASXL1*. Other frequently implicated genes are involved in growth signalling (such as *JAK2*), the DNA‐damage response pathway (*TP53*, *PPM1D* and *BRCC3*), and the spliceosome (*SF3B1*, *SRSF2* and *U2AF1*). Although the formal definition of CHIP is restricted to mutations in myeloid genes, somatic mutations also occur in genes associated with lymphoid malignancies (known as lymphoid or L‐CHIP) and in the absence of known driver mutations (also referred to as CHUD, clonal haematopoiesis with unknown drivers) (Genovese et al. 2014; Niroula et al. 2021; Weeks and Ebert 2023; Zink et al. 2017). For CHIP, and for other forms of CH, prevalence estimates depend on the sensitivity of the detection method (Danielsson et al. 2020; Dumanski et al. 2016; Lin et al. 2021; Liu et al. 2024; Thompson et al. 2019). CHIP by definition requires a VAF ≥ 2%, which does not require error‐corrected sequencing, and permits leveraging of data from large population‐based cohorts. We now know that smaller clones (down to 0.1% VAF), which can be detected with error corrected sequencing, are more frequent and become almost ubiquitous with age (McKerrell et al. 2015; Young et al. 2016). However, larger clone size is generally associated with greater risk of progression to disease, particularly haematological malignancy and, for CHIP, cardiovascular disease (Gu et al. 2023; Jaiswal et al. 2014, 2017; Lin et al. 2021; Singh et al. 2024; Weeks et al. 2023).

### 3.2 Risk Factors for Clonal Haematopoiesis

Age is a major risk factor for development of CH, and the prevalence of mLOY, mLOX, mCAs and mutation‐driven CH all increase with age (Brown et al. 2023; Gao et al. 2021; Genovese et al. 2014; Guo et al. 2020; Jacobs et al. 2012; Jaiswal et al. 2014; Jakubek, Ma, et al. 2025; Laurie et al. 2012; Lin et al. 2021; Loh et al. 2018, 2020; Machiela et al. 2016, 2015; Russell et al. 2007; Terao et al. 2020; Xie et al. 2014; Zekavat et al. 2021). Along with increased prevalence of CH, older individuals tend to have larger clone sizes, a greater number of mutations (Genovese et al. 2014; van den Akker et al. 2016; van Zeventer et al. 2021) and a different mutational spectrum including increased relative prevalence of *TET2* and spliceosome mutations (Buscarlet et al. 2017; Fabre et al. 2022; McKerrell et al. 2015; van den Akker et al. 2016). mCAs are more common in males (Gao et al. 2021; Jacobs et al. 2012; Loh et al. 2018, 2020; Machiela et al. 2015; Terao et al. 2020; Zekavat et al. 2021), although specific abnormalities have been identified that are more common in women and/or more likely to occur at a younger age (Loh et al. 2020). A clear gender predisposition is not seen for CHIP. mLOY and mCA frequency is also reported to be higher in those with European compared to African ancestry (Gao et al. 2021; Jakubek, Ma, et al. 2025; Machiela et al. 2015), whereas mLOY (Jakubek, Ma, et al. 2025) and CHIP prevalence is higher in Europeans/Whites compared to those of Hispanic or Asian ancestry (Bick, Weinstock, et al. 2020; Bolton et al. 2020; Jaiswal et al. 2014; Jakubek, Ma, et al. 2025; Terao et al. 2019).

In support of the ‘common soil of genomic instability’ hypothesis, there is overlap in the germline variants that predispose to CHIP, mLOY, mLOX and mCA (Bick, Weinstock, et al. 2020; Kar et al. 2022; Liu et al. 2024; Silver et al. 2021). For example, telomerase reverse transcriptase (TERT) is a key enzyme in telomere maintenance and is constitutively expressed in HSCs but not most other tissues of the body (Silver et al. 2021). *TERT* variants associated with longer telomeres have been linked to CHIP (Bick, Weinstock, et al. 2020; Zink et al. 2017), mLOY (Thompson et al. 2019) and mCAs (Loh et al. 2020). *TERT* variants have also been linked to diseases of ageing including cancer, cardiovascular disease and dementia, and it may be that these associations are partly mediated by CH (Silver et al. 2021).

Germline variants associated with mCAs can occur in cis (at the same locus as the mCA) or in trans (at a different region to the mCA). Interestingly, several examples have been reported where a somatic mCA in cis results in CN‐LOH of a germline alteration, and the resultant change in allelic dosage confers a proliferative advantage to the cell, consistent with the multi‐hit hypothesis of cancer development (Loh et al. 2020). As discussed further below, concurrent acquired mCAs and gene mutations can also affect the same locus (Gao et al. 2021; Saiki et al. 2021).

Some germline variants confer differential risk on different forms of clonal haematopoiesis. For example, a variant near the T cell leukaemia/lymphoma 1A (*TCL1A*) gene is associated with increased risk of *DNMT3A* CHIP (Bick, Weinstock, et al. 2020) and mLOY (Zhou et al. 2016), but reduces the risk of *TET2*‐CHIP (Bick, Weinstock, et al. 2020). Similarly, Kar et al. (2022) found a separate *TCL1A* variant as well as a variant in *SETBP1* were associated with increased risk of *DNMT3A*‐CHIP but reduced risk of mCAs. Buscarlet et al. (2017) found that *TET2*‐CHIP was more likely to occur in siblings (recurrence risk ratio 2.24 for women > 55 years) but *DNMT3A*‐CHIP showed no pattern of familial aggregation.

Environmental stressors linked to mLOY include smoking, obesity, heavy alcohol use and exposure to air pollution and polycyclic aromatic hydrocarbons (Baliakas and Forsberg 2021; Guo et al. 2020; Wright et al. 2017). Smoking has also been linked to mLOX (Liu et al. 2024), mCAs (Dawoud et al. 2020) and CHIP, particularly involving the *ASXL1* gene (Bick, Weinstock, et al. 2020; Bolton et al. 2020; Dawoud et al. 2020; Kar et al. 2022). Similarly, exposure to external beam radiotherapy (Gao et al. 2021) is a risk factor for mCAs, and chemoradiotherapy promotes expansion of CHIP clones specifically for *TP53* and other DNA damage response (DDR) pathway mutations, leading to development of therapy‐related myeloid neoplasms (Bolton et al. 2020; Coombs et al. 2017). As discussed further below, inflammation may also be an important risk factor for development or expansion of CH (Dai and Guo 2021).

### 3.3 Disease Associations

Although initially thought to be a benign age‐related phenomenon, in recent years, mLOY has been linked to increased risk of all‐cause mortality and other diseases of ageing including cancer, Alzheimer's disease and cardiovascular disease (Baliakas and Forsberg 2021; Forsberg 2017; Guo et al. 2020; Lim et al. 2024). mLOY has been associated with differences in blood counts (Jakubek, Smith, et al. 2025; Lin et al. 2020; Terao et al. 2019), but has generally not been associated with blood cancers (Forsberg 2017; Lin et al. 2021), except perhaps at high cell fraction (Ouseph et al. 2021). Very little is known about disease associations with mLOX, but it has recently been linked to increased risk of blood cancers (Brown et al. 2023; Lin et al. 2021; Liu et al. 2024), acute tonsillitis (Lin et al. 2021) and autoimmune diseases (Bianchi et al. 2012). mLOX has not been reported to increase the risk of incident non‐haematologic cancer (Liu et al. 2024). The relatively fewer disease associations with mLOX compared to mLOY may reflect the fact that mLOX is less well studied, but it is also possible that mLOX has a milder phenotype due to preferential loss of the inactive X chromosome.

Autosomal mCAs and CHIP also confer increased risk of all‐cause mortality, blood and solid organ cancer (Gao et al. 2021; Genovese et al. 2014; Jacobs et al. 2012; Jaiswal et al. 2014; Laurie et al. 2012; Loh et al. 2018, 2020; Machiela et al. 2015; Singh et al. 2024; Terao et al. 2020; Zekavat et al. 2021). mCAs overall are more commonly associated with lymphoid malignancies, but myeloid‐associated mCAs specifically predict for myeloid malignancies (Niroula et al. 2021). Similarly, myeloid CHIP increases the risk of myeloid malignancy, but lymphoid‐CHIP, which is less frequent than myeloid CHIP, is associated with increased risk of lymphoid malignancy (Bernstein et al. 2024; Niroula et al. 2021), although malignancies of the other lineage can also occur (Desai et al. 2025; Li et al. 2023; Tiacci et al. 2018) CHUD has also been linked to increased haematological malignancy (Bernstein et al. 2024; Genovese et al. 2014; Wen et al. 2024; Zink et al. 2017) and all‐cause mortality (Genovese et al. 2014; Zink et al. 2017). For both CHIP and mCAs (Loh et al. 2020; Terao et al. 2020; Zekavat et al. 2021), clone size and number of alterations are important risk factors for progression to blood cancers. Risk prediction models incorporating VAF, number of mutations, specific gene(s)/mutation(s) involved and other factors, have been developed to estimate the risk of myeloid malignancies in people with CHIP/CCUS (Gu et al. 2023; Weeks et al. 2023).

mCAs have also been associated with other diseases including dementia (Leshchyk et al. 2023), developmental delay (Jacobs et al. 2012) and a diverse range of infections (Lin et al. 2021; Zekavat et al. 2021), but overall have not been associated with cardiovascular disease (Loh et al. 2018; Saiki et al. 2021; Terao et al. 2020), with the exception of events involving the *JAK2* locus (Loh et al. 2018). By contrast, several studies support an association between CHIP and atherosclerotic disease including coronary heart disease (Bick, Pirruccello, et al. 2020; Honigberg et al. 2021; Jaiswal et al. 2017; Kessler et al. 2022), ischaemic stroke (Bhattacharya et al. 2022; Jaiswal et al. 2017) and peripheral vascular disease (Zekavat et al. 2023). The risk of cardiovascular disease with CHIP appears independent of traditional cardiovascular risk factors (including age, smoking, diabetes, blood pressure and cholesterol), and dependent on clone size, with the greatest risk being in larger clones (VAF > 10%). However, not all studies have found positive associations between CHIP and cardiovascular disease (Kar et al. 2022; van Zeventer et al. 2021). Reasons for conflicting results include methodological differences in definition and detection of CHIP as well as outcomes and adjustment factors, and potentially also inherent differences in the cohorts studied (Singh et al. 2024).

Despite being described more recently than mLOX, mLOY and mCAs, CHIP has been linked to a large variety of diseases. In addition to cancers and cardiovascular disease, CHIP has also been associated with increased risk of heart failure (Pascual‐Figal et al. 2021; Yu et al. 2021), venous thrombosis (Wolach et al. 2018), insulin resistance and diabetes (Jaiswal et al. 2014), osteoporosis (Kim et al. 2021), chronic kidney disease (Dawoud et al. 2022), gout (Agrawal et al. 2022), liver disease (Wong et al. 2023), poor performance status (Cook et al. 2019), infection (Bolton et al. 2021; Kessler et al. 2022; Vlasschaert, Akwo, et al. 2023) and others (Singh et al. 2024). Age is also a primary risk factor for neurodegenerative diseases, but little is known about their relationship with CHIP. Some studies failed to find an association between CHIP and cognitive impairment (Buizza et al. 2024; Hayden et al. 2020), whereas others surprisingly found that CHIP may be protective against the development of Alzheimer's dementia (Bouzid et al. 2023; Jakubek, Smith, et al. 2025; Matatall et al. 2025).

#### 3.3.1 Disease Associations of CHIP in Older Adults

The majority of epidemiological studies of CHIP have included only small numbers of individuals over the age of 80 years. The UK Biobank, which has been a rich resource to learn about the clinical associations of CHIP and the overlap of different forms of CH, specifically excluded people over the age of 70 years, and it is therefore uncertain if these findings can be directly applied to older adults. Three studies (Rossi et al. 2021; van den Akker et al. 2016; van Zeventer et al. 2021), including five different cohorts, have focused exclusively on the clinical implications of CHIP in those over 80 years of age. As illustrated in Table 1, the different detection methods result in quite different estimates of median VAF (4%–24%) and CHIP prevalence (6%–61%), which may have an impact on reported associations with outcomes. The earlier, smaller studies (van den Akker et al. 2016; van Zeventer et al. 2021) reported no association between CHIP and mortality in older individuals, although van Zeventer et al. (2021) did find increased all‐cause mortality in the subset of individuals with CHIP mutations in genes other than *DNMT3A* or *TET2* (HR 1.48 [95% CI: 1.06–2.08]). The lack of disease associations in early studies may reflect small sample sizes (van den Akker et al. 2016), predominance of smaller CHIP clones (van Zeventer et al. 2021) or inherent survivor bias when exclusively studying older individuals. Subsequently, Rossi et al. (2021) reported in two larger cohorts that mutation‐driven clonal haematopoiesis was associated with increased risk of all‐cause mortality, as well as myeloid blood cancers, coronary heart disease, cancer mortality and non‐cancer mortality. Importantly, people with cytopenias were not excluded from any of these studies. In the Health and Anaemia and Monizo_80+ cohorts, unexplained cytopenias occurred in 4.5% and 11.5% of the total cohorts, respectively, and those with cytopenias and mutations would be classified as CCUS rather than CHIP. Rossi et al. (2021) also developed a risk prediction model for myeloid malignancies in this older cohort, and validated it in a cohort of 75–80 year olds. Parameters associated with increased risk and included in the model were red blood cell indices (increased mean cell volume (MCV) and/or RDW), VAF (> 9.6%) and mutational pattern (co‐mutations involving *DNMT3A*, *TET2* and *ASXL1*, or spliceosome mutations).

**TABLE 1:** Association between CHIP and all‐cause mortality in studies focusing exclusively on individuals ≥ 80 years old.

Study	Cohort name	Age criteria (years)	*N*	CHIP, *n* (%)	CHIP method	Median VAF (%)	Median follow up	HR (95% CI) for overall survival	Adjustment variables
van den Akker et al. (2016)	Rotterdam study	≥ 80	646	39 (6.0)	WES	21.60	8.7 years	0.83 (0.58–1.18)	Nil
van den Akker et al. (2016)	Leiden Longevity study	≥ 89	218	40 (18.3)	WGS	23.20	9.2 years	0.94 (0.64–1.35)	Nil
van Zeventer et al. (2021)	Lifelines	≥ 80	621	382 (61.5)	ECS	4.20	6.9 years	0.91 (0.7–1.18)	Age, sex
Rossi et al. (2021)	Health & Anaemia	≥ 80	1043	342 (32.8)	Targeted	~10	NR	1.28 (1.1–1.9)	Age, sex and cytopenias
Rossi et al. (2021)	Monzino 80+	≥ 80	735	190 (25.9)	Targeted	~12	NR	1.37 (1.2–1.71)	Age, sex and cytopenias

Abbreviations: CI, confidence interval; ECS, error‐corrected sequencing; HR, hazard ratio; *N*, total number of participants; VAF, variant allele frequency; WES, whole exome sequencing; WGS, whole genome sequencing.

## 4 CH and Biomarkers of Ageing

CH reflects genomic instability, and to some extent can be considered a passive biomarker of ageing. Concordant with this, genetic variants associated with LOY are also associated with X‐chromosome loss (Brown et al. 2023; Wright et al. 2017), female‐specific cancers and later age at menopause, which is known to be a function of the ability of oocytes to detect, repair and respond to DNA damage (Thompson et al. 2019). Furthermore, there is overlap between genetic variants associated with LOY, cancer susceptibility and somatic drivers of tumour growth (Thompson et al. 2019). CHIP is linked to many diseases associated with ageing, and even non‐driver CH (CHUD) is associated with increased mortality (Genovese et al. 2014; Zink et al. 2017) or other adverse outcomes (Weinstock et al. 2024). Although CH may simply be a surrogate marker of genomic instability, there is growing evidence that CH, particularly CHIP, directly contributes to disease pathogenesis through alterations in immune or inflammatory responses. As a result, CHIP may promote phenotypes associated with ageing and correlate with older biological age.

### 4.1 Inflammation as an Instigator and Driver of CH

Inflammation is a key component of ageing and age‐associated diseases including malignancy. Chronic inflammation is known to be harmful to normal HSCs and partly responsible for the changes in haematopoiesis that accompany ageing including stem cell attrition and myeloid skewing (Trowbridge and Starczynowski 2021). However, there is evidence from in vitro and mouse models that CHIP clones have a survival advantage in the setting of inflammation, and that inflammation promotes clonal expansion (Abegunde et al. 2018; Avagyan et al. 2021; Cai et al. 2018; Heimlich et al. 2024; Hormaechea‐Agulla et al. 2021; Jakobsen et al. 2024; Meisel et al. 2018; SanMiguel et al. 2022). CHIP has also been documented to be more prevalent in people with chronic immune diseases including HIV (Bick et al. 2022; Dharan et al. 2021), autoimmune diseases (Hecker et al. 2021), inflammatory bowel disease (Cumbo et al. 2022; Zhang et al. 2019) and anti‐neutrophil cytoplasmic antibody (ANCA)‐associated vasculitis (Arends et al. 2020), perhaps reflecting the influence of inflammation on promoting expansion of CHIP clones.

### 4.2 CHIP as a Mediator of Disease Through Promoting Inflammageing

CHIP may also cause inflammation, and this may be a key pathogenic mechanism linking CHIP with non‐haematological diseases. Mouse models of *Tet2*‐clonal haematopoiesis show increased tissue expression of IL‐1B and/or downstream IL‐6, and increased atherosclerosis (Fuster et al. 2017; Jaiswal et al. 2017), heart failure (Sano, Oshima, Wang, MacLauchlan, et al. 2018; Wang et al. 2020), insulin resistance (Fuster et al. 2020), features of gout (Agrawal et al. 2022), steatohepatitis and liver fibrosis (Wong et al. 2023) and acute kidney injury (Vlasschaert et al. 2024). In many cases, it has been shown that the tissue damage can be prevented or ameliorated by blockade of the NLRP3 inflammasome or IL‐1B expression (Agrawal et al. 2022; Fuster et al. 2017, 2020; Jaiswal et al. 2017; Sano, Oshima, Wang, MacLauchlan, et al. 2018). *Tet2*‐knock out (KO) mice have also been observed to have emphysematous changes upon exposure to cigarette smoke, and upregulation of interferon signalling compared to wild type mice (Miller et al. 2022). *Dnmt3a*‐mutant models of clonal haematopoiesis have evidence of increased heart failure in response to injury, but the inflammatory cytokine profile differs from *Tet2*‐mutant models (Sano, Oshima, Wang, Katanasaka, et al. 2018). *Dnmt3a*‐deficient mice have also been shown to have reduced bone mineral density, and this is due to increased secretion of pro‐inflammatory molecules, including IL‐20, from *Dnmt3a*‐mutant macrophages that promote differentiation to osteoclasts (Kim et al. 2021).

Elevated levels of inflammatory cytokines, including IL‐6 and TNFa, have been observed in people with CHIP (Arends et al. 2023; Bick, Weinstock, et al. 2020; Böhme et al. 2022; Cook et al. 2019; Dharan et al. 2021; Kuhnert et al. 2022; Wang et al. 2022; Zhang et al. 2019). Cytokine profiles may relate to the specific CHIP gene involved; for example, a greater elevation of TNFa has been observed with *DNMT3A* CHIP (Cook et al. 2019), whereas *TET2* CHIP has been specifically associated with elevated IL‐1B (Arends et al. 2023; Bick, Weinstock, et al. 2020; Heimlich et al. 2024). A common germline variant in the IL‐6 Receptor (IL6R) gene (pAsp358Ala), which dampens IL‐6 signalling, may be associated with reduced risk of cardiovascular events (Arends et al. 2023; Bick, Pirruccello, et al. 2020; Vlasschaert, Heimlich, et al. 2023) and *Streptococcus pneumoniae* infection (Quin et al. 2024), but not haematological malignancy, in carriers of CHIP, particularly those with *TET2* mutations (Arends et al. 2023). These studies provide evidence on the role of inflammation in mediating disease phenotypes associated with CHIP, and as a potential therapeutic target. Indeed, a post hoc analysis of the CANTOS trial (Ridker et al. 2017) found that Canakinumab, a monoclonal antibody targeting IL‐1b, might reduce rates of cardiovascular events selectively in those with *TET2* mutations, but not those with other CHIP mutations or no CHIP (Svensson et al. 2022).

Overall, the above findings suggest that CHIP clones (best studied in *DNMT3A* or *TET2* mutant clones) have a survival advantage in the setting of inflammation, and that blood cells carrying these mutations have increased expression of pro‐inflammatory cytokines that (in addition to potentially favouring further expansion of CHIP clones) can cause tissue damage that at least partly accounts for the association between CHIP and diseases of ageing. Further understanding of the complex interplay between CHIP and inflammation, including for mutations other than *DNMT3A* or *TET2*, has the potential to provide novel markers to further stratify risk of disease in individuals with CHIP, as well as therapeutic targets to alter the natural history of CHIP or treat associated outcomes.

### 4.3 CH May Exacerbate Features of Normal Ageing

In addition to increased production of pro‐inflammatory cytokines, predominantly from macrophages, *Tet2*‐CHIP has also been shown to result in impaired neutrophil function including impaired phagocytosis, which may predispose to infection (Quin et al. 2024). Furthermore, differences in gene expression profiles in T cells derived from individuals with CH indicate that CH may be associated with impaired T cell function (Heimlich et al. 2024). Consequently, CHIP is associated with impaired innate and adaptive immune cell function (immunosenescence), as well as increased production of pro‐inflammatory cytokines (inflammageing), both of which occur as a part of normal haematopoietic ageing, and may account for the increased predisposition to many diseases of ageing in people with CHIP. Similarly, myeloid skewing, a key feature of ageing HSCs, has also been observed in experimental models of CHIP (Buscarlet et al. 2018; Fuster et al. 2020; Izzo et al. 2020; Jakobsen et al. 2024). Red cell width (RDW), which is a measure of the variation in size of red blood cells, increases with age (Hoffmann et al. 2015), and has been proposed as a biomarker of ageing as it is associated with increased risk of mortality and diseases including cardiovascular disease and cancers (Patel et al. 2010; Pilling et al. 2018). RDW is elevated in people with CHIP (Bick, Weinstock, et al. 2020; Jaiswal et al. 2014; Kar et al. 2022), and it is unknown whether the links between RDW and diseases of ageing may be mediated by the unrecognised presence of CHIP. Jakobsen et al. (2024) recently demonstrated that both mutant and non‐mutant stem cells from individuals with CH, when compared to wild type cells from those without CH, are enriched for transcriptional signatures associated with ageing stem cells. These observations suggest that CHIP may exacerbate features of normal ageing such that people with CHIP appear phenotypically older. Consistent with this, CH has also been linked to other biomarkers of ageing including DNA methylation‐based epigenetic clocks and telomere length.

### 4.4 CH and DNA Methylation

Methylation of cytosine‐phosphate‐guanine (CpG) dinucleotides to form 5‐methylcytosine (5mC) is an epigenetic modification that can influence gene transcription and has a key role in differentiation and development. DNA methylation levels vary among different cells and tissues, and can be altered in disease states, as well as by genetic and environmental influences. DNA methylation patterns change with age, and several groups have identified specific CpG sites that are either hypo‐ or hyper‐methylated with age. Combinations of sites have been used to develop ‘epigenetic clocks’, which can predict a person's age and can thus be used as a biomarker of ageing. As methylation profiles are influenced not only by chronological age but also various other genetic and environmental influences (including diet, smoking and BMI), epigenetic clocks may be able to capture elements of ‘biological age’. Indeed, epigenetic clocks can predict, to varying degrees, overall mortality as well as diseases associated with ageing including cardiovascular disease, cancers, neuropathology, frailty and physical functioning (Horvath and Raj 2018; Quach et al. 2017; Simpson and Chandra 2021).

Little is known about the relationship between mCA, mLOX or mLOY and methylation profiles or epigenetic ageing. However, the two most commonly mutated genes in CHIP (*DNMT3A* and *TET2*) are involved in DNA methylation, with *DNMT3A* having an important role in de novo methylation and *TET2* in demethylation by catalysing the oxidation of 5‐methylcytosine (5mC) to 5‐hydroxymethylcytosine (5‐hmC) (Schoofs et al. 2014). CHIP mutations, particularly in *DNMT3A* and *TET2*, may therefore result in aberrant methylation profiles with consequences for cellular processes or epigenetic age measurements.

#### 4.4.1 Altered Methylation Profiles in CHIP

Aberrant methylation profiles have been well documented in *DNMT3A‐* and *TET2‐*mutant acute myeloid leukaemia (AML) (Schoofs et al. 2014), and more recently in CHIP. Small early studies reported evidence of hypermethylation in people with *TET2‐*CHIP compared to those without (Buscarlet et al. 2017; Busque et al. 2012). Epigenome wide association studies have revealed that specific CpG sites can be identified that are differentially methylated in *DNMT3A*‐ or *TET2*‐CHIP compared to non‐CHIP controls, with little overlap between the sites implicated for *DNMT3A* versus *TET2* (Tulstrup et al. 2021; Uddin et al. 2022). Differentially methylated sites identified in *DNMT3A*‐CHIP or *TET2*‐CHIP form a subset of sites that are differentially methylated in AML with the respective mutation (Tulstrup et al. 2021; Uddin et al. 2022). Consistent with the biological role of *DNMT3A* in de novo methylation and *TET2* in de‐methylation, differentially methylated sites (particularly those replicated between cohorts) in *DNMT3A‐*CHIP were more likely to be hypomethylated (Kuhnert et al. 2022; Tulstrup et al. 2021; Uddin et al. 2022) whereas those in *TET2‐*CHIP were more likely to be hypermethylated (Tulstrup et al. 2021; Uddin et al. 2022). Hypermethylation in *TET2‐*CHIP has also been shown to correlate with VAF (Buscarlet et al. 2017; Tulstrup et al. 2021; Uddin et al. 2022). It has also been suggested that methylation‐based models may be able to predict *TET2* mutation status (Tulstrup et al. 2021). Functional implications of altered methylation are discussed further in Section 4.4.3.

#### 4.4.2 CHIP and Epigenetic Age Acceleration

DNA methylation‐based epigenetic clocks aim to reflect biological age and have been correlated with diseases of ageing. Several different epigenetic clocks have been published including the Hannum, Horvath, DNAmPhenoAge and DNAmGrimAge. These differ in the number and specific CpG sites used, how the CpGs were selected (correlation with chronological age and/or other outcome variables), the tissues from which they are derived and for which they have been validated, and their ability to predict for mortality or disease (Hannum et al. 2013; Horvath 2013; Horvath and Raj 2018; Levine et al. 2018; Lu et al. 2019). Two additional measures frequently used are intrinsic‐ and extrinsic‐ epigenetic age acceleration (IEAA and EEAA). ‘Epigenetic age acceleration’ (i.e., older epigenetic age than chronological age) is a measure that reduces the confounding effect of chronological age, and is correlated with mortality and several diseases. IEAA and EEAA are age acceleration residuals calculated from the intrinsic Horvath or extrinsic Hannum clocks, respectively, after either minimising (for IEAA) or emphasising (for EAAA) the impact of age‐associated changes in blood cell composition (e.g., lymphopenia). As a result, IEAA is thought to reflect cell‐intrinsic ageing and EAAA reflects the impact of ageing of the immune system or immunosenescence (Horvath and Raj 2018; Quach et al. 2017).

The first study to systematically examine the relationship between CHIP and epigenetic age was performed by Robertson et al. (2019) in 1136 individuals in the Lothian Birth Cohort (LBC) 1921 and 1936. The presence of CHIP overall was associated with an increase in IEAA, with a greater age acceleration for *TET2* mutations compared to *DNMT3A* mutations. This finding is consistent with several studies showing that *TET2‐*CHIP has a greater correlation with disease risk than *DNMT3A‐*CHIP (Singh et al. 2024; Weeks et al. 2023). Age acceleration using different epigenetic clocks showed variable results, with all the clocks showing an association with CHIP and age acceleration in the older cohort (LBC1921), although with varying effect sizes. The association between CHIP and accelerated epigenetic age was also confirmed by a second and larger study of 5522 individuals within the Trans‐Omics for Precision Medicine (TOPMed) cohort (Nachun et al. 2021). Again, CHIP was associated with increased age acceleration, this time reaching significance for extrinsic as well as intrinsic age acceleration. A third study of 308 older Danish twins also showed similar trends (Soerensen et al. 2022). Most recently, accelerated epigenetic ageing, particularly in the extrinsic clocks, has been linked to a faster estimated expansion rate of CHIP clones in a cohort of 297 individuals (Mack et al. 2024).

Larger VAFs (Nachun et al. 2021; Soerensen et al. 2022) and greater number of mutations (Nachun et al. 2021) show increasing association with epigenetic age acceleration, and these associations are strongest with extrinsic clocks (Hannum and EAAA). Both Nachun et al. (2021) and Soerensen et al. (2022) reported that *TET2* was more strongly associated with extrinsic age acceleration, whereas *DNMT3A* was associated with intrinsic age acceleration. This divergence has been hypothesised to reflect differing mechanisms of clonal expansion, where intrinsic stem cell proliferation rate may drive *DNMT3A*‐CHIP whereas extrinsic signals from an ageing microenvironment may be more important for *TET2*‐CHIP expansion, which is known to develop later in life (Soerensen et al. 2022). Leveraging the twin nature of their cohort to account for the effects of genetic and environmental confounders, Soerensen et al. (2022) conducted intra‐pair analyses limited to twins with discordant CHIP status. In this analysis, *TET2* status showed a consistent association with EEAA but there was no longer an association between *DNMT3A* and IEAA, suggesting the previously observed associations may be due to unmeasured confounding. Mutations in other genes, including the DNA damage response pathway (DDR) genes (*TP53*, *PPM1D* and *BRCC3*), have not been specifically associated with increased age acceleration (Nachun et al. 2021).

The presence of epigenetic age acceleration may help further risk‐stratify people with CHIP, although results have been conflicting. Nachun et al. (2021) defined individuals as AgeAccelHG+ if they showed evidence of age acceleration in both of two orthogonal epigenetic clocks, Hannum and GrimAge. Both CHIP and AgeAccelHG+ were associated with increased risk of coronary heart disease and all‐cause mortality (although the results did not reach statistical significance (*p* = 0.077) for CHIP and all‐cause mortality), and the risk was highest in the 40% of CHIP carriers who also had age acceleration (CHIP+/AgeAccelHG+). In fact, individuals with CHIP who did not have age acceleration (CHIP+/AgeAccelHG−) had a similar risk to those without CHIP (CHIP−/AgeAccelHG−), suggesting that AgeAccelHG was a more important predictor of outcomes than CHIP status in this cohort. However, the poorer outcomes with concurrent CHIP and AgeAccelHG were not replicated in the study of older Danish twins, where there was no increase in the risk of all‐cause mortality in this group compared to those that were CHIP−/AgeAccelHG− (Soerensen et al. 2022). There were several differences between the two studies, including in population characteristics and CHIP measurement, which may contribute to the conflicting results. For example, Soerensen et al. (2022) studied twins enrolled from age 70 years whereas the TOPMed cohorts (Nachun et al. 2021) were younger and one of the cohorts was over‐sampled for incident stroke and venous thrombo‐embolism. Furthermore, CHIP status was determined with an error‐corrected sequencing assay compared to whole genome sequencing in TOPMed, so smaller clones were more represented among the twin population. It may be that the risk‐stratification with epigenetic age among people with CHIP is of greater relevance in a population with underlying cardiovascular disease or comorbidities, or in a younger cohort, or in those with larger CHIP clones. Further studies are required to assess the impact of concurrent CHIP and epigenetic age acceleration on clinical outcomes, and the factors that may influence this. It also remains to be determined why and how some people with CHIP have accelerated epigenetic ageing, and it may be that this results from interactions between CHIP and other genetic or environmental factors, making the teasing out of the direct or indirect contribution of CHIP to disease even more challenging.

#### 4.4.3 Altered Methylation: Cause or Coincidence?

It remains unknown whether altered DNA methylation profiles with age have a mechanistic role in driving ageing‐related tissue or organ dysfunction, or whether they are a passive biomarker of ageing. Although some of the CpG sites used in epigenetic clocks may have little or no contribution to biological ageing, there is evidence to support the former hypothesis that altered methylation profiles contribute to biological ageing. For example, DNAmPhenoAge acceleration is associated with activation of proinflammatory pathways as well as downregulation of DNA damage response pathways (Levine et al. 2018), both of which are important hallmarks of ageing. There is also growing evidence that epigenetic alterations, such as DNA methylation, may have a direct role in regulating inflammatory responses that lead to pathologies such as cardiovascular disease, cancers and neurological disorders, in response to external stimuli or lifestyle factors such as smoking, obesity, diet and physical activity (Ramos‐Lopez et al. 2021).

Altered methylation profiles may have a mechanistic role in disease pathogenesis mediated by *DNMT3A*/*TET2* mutations. Methylation profiles are important for differentiation of haematopoietic stem cells. Disruption of methylation profiles due to *Dnmt3a*‐ or *Tet2*‐mutations results in increased self‐renewal (Challen et al. 2011; Moran‐Crusio et al. 2011) and skewing of differentiation towards erythroid cells in *Dnmt3a* deficiency and myelomonocytes in *Tet2* deficiency (Izzo et al. 2020). In comparing the methylation profiles of HSCs and their progeny, Uddin et al. (2022) observed that the differentially methylated sites associated with *TET2*‐CHIP, which were generally hypermethylated, were fully methylated in HSCs and those associated with *DNMT3A*‐CHIP had lower levels of methylation in HSCs. Hence, the methylation profiles of mature CHIP cells more closely resemble methylation profiles in HSCs, which might potentially promote self‐renewal and explain how CHIP mutations confer a proliferative advantage to cells.

Downstream consequences of altered methylation profiles in CHIP may be due to altered binding of specific transcription factors (Izzo et al. 2020; Tulstrup et al. 2021; Uddin et al. 2022). Indeed, it has been shown that sites that are differentially methylated in *DNMT3A‐* and *TET2*‐CHIP are enriched in binding motifs of transcription factors involved in haematopoiesis (Tulstrup et al. 2021; Uddin et al. 2022). Just as cancer‐associated differentially methylated regions (C‐DMR) have been shown to be depleted in CpG islands (CGI) and enriched in surrounding areas, differentially methylated sites associated with *DNMT3A*‐ or *TET2*‐CHIP were depleted in CGIs but enriched in CpG shores (≤ 2 kb from CGI) and shelves (2–4 kb from CGI), respectively. CpG sites associated with *DNMT3A*‐CHIP were also enriched for sites that have been implicated as C‐DMR (Uddin et al. 2022). In gene ontology enrichment analyses, sites differentially methylated in *DNMT3A*‐CHIP were associated with genes involved in development and cellular processes (Uddin et al. 2022), whereas sites that were differentially methylated in *TET2*‐CHIP were associated with immune processes (Tulstrup et al. 2021; Uddin et al. 2022). Uddin et al. (2022) also demonstrated that the differential methylation profiles of *DNMT3A* and *TET2* had cell‐specific effects with *DNMT3A*‐linked sites being in regions associated with HSCs whereas *TET2*‐associated hypermethylation occurred more in regions of importance in mature blood cells including monocytes. In a small cohort of people with COPD, *DNMT3A*‐mediated hypomethylation was shown to be associated with increased pro‐inflammatory cytokines (IL‐6 and TNFa) (Kuhnert et al. 2022). Mendelian Randomisation (MR) analyses have also suggested that CHIP‐associated CpGs, which are linked to genes involved in lipid metabolism, inflammation and atherosclerosis, are causally associated with altered risk of coronary artery disease (Uddin et al. 2022).

Overall, CHIP mutations, particularly in *DNMT3A* and *TET2*, may cause altered methylation profiles that have a direct impact on epigenetic age acceleration as well as cellular processes including haematopoietic cell proliferation and differentiation, and promoting inflammation.

### 4.5 CH and Telomere Length

Telomeres are repetitive DNA sequences found at the end of chromosomes that shorten with each cell division and on exposure to oxidative stress. When a critically short length is reached, the cell exits the cell cycle and becomes senescent (Rossiello et al. 2022; Sanders and Newman 2013). Accumulation of senescent cells and the resultant pro‐inflammatory senescence‐associated secretory phenotype (SASP) promotes ageing and age‐associated diseases (Rossiello et al. 2022). Telomere length is maintained in cells by the telomerase enzyme, homologous recombination during mitosis and chromosomal end‐joining (Sanders and Newman 2013). Leucocyte telomere length can be measured in many ways, including Southern blotting, qPCR‐based assays and with fluorescence in situ hybridisation (FISH), but telomere length can also be estimated or imputed from genomic data or from methylation profiles (Nachun et al. 2021; Nakao et al. 2022; Rossiello et al. 2022; Zink et al. 2017). Shorter telomeres have been associated with older age and male sex and, albeit inconsistently, with mortality and other age‐related traits or diseases including cardiovascular disease and cancers (Horvath and Raj 2018; Jylhävä et al. 2017; Rossiello et al. 2022; Sanders and Newman 2013). Furthermore, diseases such as congenital dyskeratosis and Werner's syndrome, which are caused by mutations in genes involved in telomere maintenance, share phenotypic characteristics of accelerated ageing (Sanders and Newman 2013). Telomere length has therefore been proposed as a biomarker of ageing (Horvath and Raj 2018; Jylhävä et al. 2017; Sanders and Newman 2013).

The relationship between CH and telomere length is complex, and has been proposed to be bidirectional. On the one hand, mLOX (Liu et al. 2024), mLOY (Jakubek, Ma, et al. 2025; Wang et al. 2024), mCAs (Brown et al. 2023) and CHIP (Buscarlet et al. 2017; Nachun et al. 2021; Nakao et al. 2022; Zink et al. 2017) have all been associated with shorter leucocyte telomere length independent of age. Number of mutations (Brown et al. 2023; Nachun et al. 2021; Nakao et al. 2022) and higher VAF (Brown et al. 2023; Nakao et al. 2022) or cellular fraction of mLOY (Jakubek, Ma, et al. 2025) or mCA (Brown et al. 2023) correlate with shorter telomere length. Germline variants in genes implicated in telomere maintenance (such as *TERT*) have been strongly linked to CHIP, mLOY and mCAs; however, the highest risk alleles for CHIP tend to predict for longer rather than shorter telomeres (Bick, Weinstock, et al. 2020; Loh et al. 2020; Silver et al. 2021; Terao et al. 2020; Thompson et al. 2019; Zink et al. 2017). Concordant with this, germline variants in *TERT* that predict for longer telomeres have also been linked to increased epigenetic age (Lu et al. 2018). A small study of 12 carriers of *POT1* (protection of telomeres 1) mutations, which are known to be associated with longer telomeres, had a ten‐fold higher prevalence of CHIP compared to non‐carrier relatives who also had shorter telomeres (DeBoy et al. 2023). Longer genetically‐predicted telomere length has also been linked to increased risk of CHUD (Wen et al. 2024), and to mCAs (Brown et al. 2023, 2020), whereas shorter genetically‐predicted telomere length is associated with mLOY (Brown et al. 2023; McLoughlin et al. 2025). A hypothesis from Mendelian randomisation studies that potentially explains the contradictions for CHIP is that longer telomere length predisposes to CH (Kar et al. 2022; Nakao et al. 2022), possibly by allowing more opportunities for acquisition of mutations, as well as supporting the expansion of clonal populations that would otherwise be lost with telomere shortening (DeBoy et al. 2023). However, CH then causes shortened telomeres perhaps due to increased cell proliferation (Nakao et al. 2022). Importantly, Nakao et al. (2022) found that shorter telomere length and CHIP were independently associated with increased risk of incident coronary artery disease, and propose that the effect of CHIP on coronary artery disease risk may be partly mediated by telomere shortening. This hypothesis has not yet been examined in any other studies, and it remains unknown whether the interaction between CHIP and telomere length has an impact on other phenotypes or may be a pathogenic mechanism in CHIP. McLoughlin et al. (2025) recently proposed that telomere attrition may be an important mechanism driving clonal expansion of spliceosome mutations. They demonstrated that shorter genetically‐predicted telomere length was causally linked with spliceosome mutations, in contrast to other more common forms of CHIP (such as *DNMT3A* and *TET2*) that were linked to longer genetically‐predicted telomere length as discussed above. Measured leukocyte telomere length was also shorter in people with spliceosome or *PPM1D* mutations; however, telomeres became longer with increasing *SRSF2* VAF, in contrast to further shortening with *PPM1D* or the more common *DNMT3A* or *TET2* mutations. They proposed that spliceosome mutations may prevent critical telomere shortening, and thus provide a fitness advantage to cells. Whether targeting telomere attrition will modify expansion of spliceosome clones or disease phenotypes associated with CHIP remains to be explored (McLoughlin et al. 2025).

## 5 Intersections of Different Types of CH

Apart from mLOX and mLOY which occur exclusively in females or males, respectively, the different forms of CH are not mutually exclusive and may co‐exist, either within the same clone or within distinct clones within the same individual. The differing detection techniques have meant that, until recently, little was known about the co‐existence of different forms of CH or the impact of this on clonal expansion or risk of disease. The adaptation of genotyping arrays or sequencing to detect chromosomal alterations has facilitated the detection of these abnormalities in large datasets, and a growing understanding of the overlap between different forms of CH.

### 5.1 mLOX and mCAs or CHIP

Very little is known about the overlap between mLOX and either mCAs or mutation‐driven CH. In a systematic analysis of different forms of clonal haematopoiesis in the UK biobank, Brown et al. (2023) found an association between mLOX and mCAs but not CHIP. It is likely that the concurrent presence of mLOX with mCAs confers a higher risk of haematologic malignancy than either mLOX or mCA alone (Brown et al. 2023; Liu et al. 2024). For example, in the analysis by Brown et al. (2023), the risk of lymphoid malignancy was increased with isolated mLOX (HR 1.46 [95% CI: 1.06–2.01]) or mCA (HR = 5.24 [4.47–6.4]), but the risk was higher when both were present concurrently (HR = 10.93 [6.98–17.13]), and even higher if CHIP was present as well (HR = 41.1 [18.34–92.09]). Similarly, although mLOX alone or in combination with mCAs was not associated with myeloid malignancy, the presence of mLOX in addition to both mCAs and CHIP conferred a higher risk of myeloid malignancy than CHIP or mCA alone or in combination.

### 5.2 LOY and mCAs or CHIP

After accounting for the confounding effect of age, Jakubek, Ma, et al. (2025) reported no association between the overall prevalence of mCAs and mLOY, although there may be positive or negative associations with specific mCAs.

Few studies have examined the frequency and clinical implications of co‐occurrence of CHIP with LOY. Initial studies had suggested that the prevalence of point mutations was greater in those with mLOY at cell fractions ≥ 10% (Dawoud et al. 2023; Kamphuis et al. 2023; Ljungström et al. 2022; Zink et al. 2017), but not if mLOY is only present at lower levels (Dawoud et al. 2023; Kamphuis et al. 2023), with stronger associations at higher cell fractions (Dawoud et al. 2023; Ouseph et al. 2021). However, two recent studies in independent cohorts (the UK biobank and a subset of TOPMed) have reported an inverse relationship between LOY and CHIP, specifically DNMT3A‐CHIP (Brown et al. 2023; Jakubek, Ma, et al. 2025). Additionally, both also reported an inverse association between CHIP VAF and mLOY, suggesting that the two processes may compete against each other for clonal dominance. By contrast, Zhang et al. (2022) proposed that mLOY itself can promote genomic instability and mLOY at a cellular level may facilitate development of somatic mutations. This may be particularly relevant for larger mLOY clones, where shorter telomere length may be a mechanism promoting genomic instability and acquisition of additional point mutations (Jakubek, Ma, et al. 2025). Consistent with this hypothesis, others have reported a positive correlation between clone sizes for point mutations and LOY, suggesting that they have a tendency to co‐exist in the same clone (Dawoud et al. 2023; Ljungström et al. 2022). Furthermore Kamphuis et al. (2023) reported higher clone sizes in those with concurrent mLOY and gene mutations, supporting a model of greater fitness when the two forms of CH are present together. Ouseph et al. (2021) also reported that higher mLOY cell fraction (> 75% compared to < 25%) was more likely to be associated with somatic mutations at a higher VAF, as well as with multiple mutations. Overall, these studies report conflicting findings regarding the co‐occurrence of LOY and CHIP, which may relate to methodological differences in the detection of LOY and CHIP, particularly the sensitivity to smaller clone sizes. Additionally, it may be that the effect of co‐occurrence is influenced by whether the LOY and CHIP are in the same or competing clones, which is not easy to determine from bulk sequencing data, as well as the specific CHIP mutations involved.

Both mLOY and CHIP have been linked with increased risk of all‐cause mortality, malignancies, cardiovascular disease and other pathologies; however, it remains to be determined how the two might interact together to influence risk when present concurrently. Kamphuis et al. (2023) investigated the co‐occurrence of mLOY and myeloid gene mutations in 538 men in the Netherlands. In this study, gene mutations conferred an increased risk of all‐cause mortality but mLOY did not, and there was no evidence of an interaction between the two forms of CH. Importantly, the median cell fraction for mLOY was 5.8%, well below the 10% cut‐off used to define mLOY in other studies. It remains unknown whether this would be the same if considering mLOY at a cell fraction of ≥ 10%. Brown et al. (2023) also reported no impact of mLOY on incident lymphoid or myeloid malignancy in the UK biobank, and little indication of a significantly higher risk when mLOY was present in conjunction with mCAs or CHIP, compared to either mCA or CHIP alone. Overall, limited available evidence suggests concurrent mLOY adds little to the risk of haematological malignancy associated with CHIP or mCAs, but cellular fraction may be an important consideration and has not been well studied.

### 5.3 CHIP and mCAs

Bick, Weinstock, et al. (2020) reported that mCAs and CHIP detected by whole genome sequencing (WGS) did not occur more frequently than expected by chance in a subset of people from the TOPMed cohort. However, subsequent studies in people with solid tumours (Gao et al. 2021), as well as participants from the UK (Brown et al. 2023; Niroula et al. 2021) and Japanese (Saiki et al. 2021) biobanks using targeted or whole exome sequencing (WES) to identify CHIP, have reported that mCAs are more common in those with gene mutations compared to those without, even after accounting for age. The frequent co‐occurrence may result from shared risk factors (‘common soil’), particularly germline variants, which overlap significantly between these two forms of CH (Kar et al. 2022), or potentially a synergistic relationship between them. In support of the hypothesis that mCAs and mutation‐driven CH together confer greater clonal fitness, their co‐occurrence is associated with larger clone size (Brown et al. 2023; Gao et al. 2021; Saiki et al. 2021), although this may be related to the total number of clonal alterations rather than the co‐occurrence of mCAs and mutations per se (Saiki et al. 2021). As described below, mCAs and mutations can affect the same genomic locus, and such cases demonstrate even higher clone sizes than concurrent but non‐overlapping mCAs and mutations (Brown et al. 2023). In most overlapping cases, the VAF of CHIP clones tends to be higher than the mCA cell fraction, suggesting the CHIP mutation occurred first (Brown et al. 2023).

Frequent co‐occurrence with mCAs has been observed for the common CHIP genes including *DNMT3A*, *TET2*, *ASXL1, TP53*, *JAK2*, *SF3B1* and *SRSF2* (Brown et al. 2023; Gao et al. 2021; Saiki et al. 2021). People with mCAs, compared to those without, are particularly more likely to have mutations in *MPL*, *JAK2* and *EZH2* (Gao et al. 2021; Niroula et al. 2021; Saiki et al. 2021). Gene mutations and mCAs (typically CN‐LOH) affecting the same locus are also more frequent than expected by chance (Brown et al. 2023; Saiki et al. 2021), and result in homozygosity of the mutant allele (Brown et al. 2023; Gao et al. 2021; Niroula et al. 2021; Saiki et al. 2021). This is analogous to the multi‐hit pathogenesis evolving from germline mutations (Loh et al. 2018, 2020; Terao et al. 2020). Examples include co‐mutations involving *JAK2* V617F and 9p CN‐LOH, *DNMT3A* and 2p deletion or CN‐LOH, and *TP53* and 17p CN‐LOH (Gao et al. 2021; Niroula et al. 2021; Saiki et al. 2021). These co‐mutations have all been identified in patients with overt myeloid disorders. Similarly, there are also recurrent patterns of mCAs and mutations affecting different loci and reflecting those seen in haematological malignancies, such as 4q24 (which harbours the *TET2* locus) frequently co‐occurring with *SRSF2* mutations, as occurs in myeloid malignancies. These patterns of co‐mutations not only show that characteristic genetic changes can precede overt malignancy (as most people with recurrent co‐mutations did not have significant blood count abnormalities) and support a multi‐hit pathway for cancer development, but also highlight potential mechanisms of pathogenesis (Gao et al. 2021). In the Biobank Japan study, co‐occurrence at the same locus was an independent risk factor for death from haematological malignancy, regardless of the total number of mutations, but did not influence the risk of cardiovascular mortality (Saiki et al. 2021).

All these studies report increased risk of haematological malignancy (or death from haematologic malignancy) with concurrent gene mutations and mCAs compared to either change alone (Brown et al. 2023; Gao et al. 2021; Niroula et al. 2021; Saiki et al. 2021). For example, Gao et al. (2021) reported the cumulative 3‐year incidence of leukaemia was < 1% for those with either gene mutations or mCA, but 14.6% for those with both. Other risk factors for haematologic malignancy were the number of somatic alterations, clone size, as well as the specific alterations involved (Gao et al. 2021). Weeks et al. (2023) reported a 10‐year cumulative incidence of myeloid neoplasm of 1.23% and 2.76% for those with CHIP or myeloid‐mCAs, respectively, but 18.3% in those with both abnormalities. In each of the clonal haematopoiesis risk score (CHRS) groups, the risk of myeloid neoplasms was higher in those with mCAs (Weeks et al. 2023). In addition to increasing the risk of death from haematological malignancy, co‐occurrence of gene mutations at VAF > 5% with copy number alterations in the Biobank Japan cohort was also associated with increased risk of cardiovascular and all‐cause mortality compared to either lesion alone (Saiki et al. 2021).

## 6 Conclusion and Future Directions

The different forms of CH share common germline and environmental risk factors and have overlapping prevalence and disease associations, suggesting they reflect common underlying processes of ageing. CHIP is also associated with other biomarkers of ageing, namely accelerated epigenetic age and shorter telomere length. The presence of CHIP may reflect a biologically older haematopoietic system and exacerbate features of normal ageing, including inflammageing and immunosenescence, which may be important causal mechanisms explaining the association between CHIP and a variety of diseases of ageing. Additionally, inflammation likely also promotes further expansion of CHIP. Different forms of CH may work together to promote clonal expansion and synergistically promote disease including through promoting inflammation. CH may also synergise with, or be influenced by, other sources of inflammation outside the haematopoietic system, potentially including somatic mutations in other tissues or epigenetic changes. There is some evidence that different forms of CH may make independent contributions to disease risk. For example, concurrent presence of CHIP and mCAs confers a higher risk of leukaemia than either form of clonal haematopoiesis alone, and one study has suggested that concurrent CHIP and epigenetic age acceleration may confer a higher risk of mortality and coronary heart disease than either marker alone. Further studies are required to confirm the frequency and clinical implications of co‐occurrence of different forms of clonal haematopoiesis and other biomarkers of ageing. Animal models with combinations, or models with concurrent mutations in the same cell, may also provide insights into mechanisms of clonal expansion and disease, as well as providing putative targets for prevention.

## Author Contributions

J.S., E.M.W., D.J.C. and Z.K.M. designed the review. J.S. drafted the initial manuscript. All authors critically reviewed the manuscript.

## Funding

J.S. is supported by a HSANZ/Leukaemia Foundation PhD New Investigator scholarship.

## Conflicts of Interest

The authors declare no conflicts of interest.

## Data Availability

Data sharing not applicable to this article as no datasets were generated or analysed during the current study.
